# Low birth weight and renal consequences: knowing about it means preventing it

**DOI:** 10.1590/2175-8239-JBN-2023-E013en

**Published:** 2023-12-11

**Authors:** Maria Cristina de Andrade, Nilzete Liberato Bresolin, Ana Paula Brecheret

**Affiliations:** 1 Universidade Federal de São Paulo, Escola Paulista de Medicina, São Paulo, SP, Brazil.; 2 Universidade Federal de Santa Catarina, Centro de Ciências da Saúde, Florianópolis, SC, Brazil.

Premature birth is a significant public health problem due to its implications in neonatal morbidity and mortality, and its complex etiology makes it difficult to establish prevention actions and treat its complications^1^. The main maternal causes include pregnancy complications (pre-eclampsia/eclampsia, maternal hemorrhage, placental abruption, and placenta previa), comorbidities such as diabetes and other chronic diseases that precede pregnancy, as well as kidney or heart diseases^2^. Environmental factors such as exposure to pollution, stress, drugs, toxic agents, and nutritional problems seem to influence changes in the disease development and progression^3^.

Premature newborns (NB) have altered nephrogenesis, and the reduction in the number of nephrons, which is aggravated by intrauterine growth restriction (IUGR), appears to exacerbate nephron loss. To date, few studies have investigated the role of reduced nephron number concerning neonatal kidney impairment^4^.

In a recently published meta-analysis, Yu et al.^5^ confirmed the inverse association between birth weight and chronic kidney disease (CKD), observed in previous studies, and revealed the shared maternal genetic basis between low birth weight and CKD, and the direct fetal and indirect maternal causal effects of birth weight that can lead to this negative factor^5^.

In addition to CKD, several studies confirm the association between low birth weight (LBW), premature birth, and acute kidney injury (AKI). In a meta-analysis of 50 papers involving more than 10 thousand premature and low birth weight newborns, Wu et al.^6^ evaluated the incidence and impact of AKI; its incidence was 25%, and those with AKI had a significantly higher risk of death. In the “Renal deficit and associated factors in children born with low birth weight” study, the authors demonstrate that NB with LBW have a higher prevalence of impaired kidney function, which can be aggravated using nephrotoxins and reinforce the need to evaluate kidney function in these children^7^.

Drug-induced nephrotoxicity is a frequent and underdiagnosed cause of AKI in the neonatal period. In a study with 107 premature infants with birth weight less than 1500g, Rhone et al.^8^ reported that 86% of patients were exposed to at least one nephrotoxic medication, the most frequent of which were gentamicin and indomethacin, and a quarter of patients presented AKI. In this sample, none of the patients who did not receive nephrotoxic medications had AKI.

Newborns with intrauterine growth restriction are born with fewer functioning nephrons and premature babies do not complete nephrogenesis at birth; In both cases, treatment with nephrotoxic medications can cause a further decrease in the number of nephrons. Experimental data showed a clear association between exposure to nephrotoxic medications and alterations in nephrogenesis^8,9^.

Nephrotoxic medications received during fetal life and during postnatal nephrogenesis can interfere with the development of nephrons, contributing to further increasing the risk of adult-onset chronic kidney disease in children born prematurely^10^.

As a preventive measure, systematic surveillance of exposure to nephrotoxic medications and continuous risk assessment for AKI in this population can help reduce the incidence of neonatal AKI, and CKD in the long term.

There has been great interest in the relationship between fetal development and the emergence of chronic non-communicable diseases (NCDs) in recent years. Terminology such as fetal programming, developmental origins of health and disease (DOHaD), and epigenetic factors have been coined. NCDs include cardiovascular diseases, type 2 diabetes mellitus, hypertension, dyslipidemia, proteinuria, and CKD, which can be programmed during the early stages of fetal development and manifest later, when there is an additional impact from lifestyle and other acquired habits, which are risk factors that interact with genetic factors^11^.

According to Barker’s hypothesis, different forms of NCDs originate from the “development of plasticity” in response to malnutrition during fetal life and childhood. The development of plasticity gives living things the ability to adapt to the environment within a single generation, as well as undergo adaptations over many generations through the natural selection of genes. Later, Brenner et al. proposed that the reduction in the number of nephrons, resulting from IUGR or prematurity, could contribute to hypertension by limiting sodium excretion due to a reduced filtration surface area, which could increase the risk of CKD by reducing renal adaptive capacity if other nephrons are lost due to injury^11^.

According to Barker’s theory, although NCDs may have an intrauterine origin, they can be modified by postnatal growth and living conditions. In the Helsinki study, children born with low birth weight who developed hypertension showed an acceleration in weight gain and body mass index (BMI); however, if these children’s BMI had been average or lower, there would have been little change in the incidence of hypertension^11^. In the “Renal deficit and associated factors in children born with low birth weight” study, the authors demonstrated that the current normal weight of children was a protective factor against the risk of renal function deficit^7^.

Therefore, monitoring blood pressure, weight, renal function, microalbuminuria, and proteinuria, as well as running kidney ultrasound scans in these children, with an emphasis on preventive measures, is necessary to reduce the risk of cardiovascular and kidney diseases.
